# Role of social network in decision making for increasing uptake and continuing use of long acting reversible (LARC) methods in Pakistan

**DOI:** 10.1186/s12978-021-01149-0

**Published:** 2021-05-17

**Authors:** Mariyam Sarfraz, Saima Hamid, Patrick Rawstorne, Moazzam Ali, Rohan Jayasuriya

**Affiliations:** 1 Health Services Academy, Islamabad, Pakistan; 2 Fatima Jinnah Women University, Rawalpindi, Pakistan; 3 School of Public Health and Community Medicine, University of New South Wales, Sydney, Australia; 4 Department of Sexual and Reproductive Health, World Health Organization, Geneva, Switzerland

**Keywords:** Long acting reversible contraceptives, Contraceptive use decision making, Social networks, Pakistan, Married women, Family planning, Birth spacing

## Abstract

**Introduction:**

Despite evidence from recent Demographic Health Surveys that show 98% of the adult Pakistani population have an awareness of at least one modern contraceptive method, only 25% of married couples in Pakistan used a modern method of contraception. Of the modern contraceptive methods, LARC usage has increased only from 2.1 to 3%. This low uptake is puzzling in the context of high awareness of LARC methods and its availability through public sector facilities at subsidized costs. This study aimed to understand the social influences in initiating and continuing use of an LARC methods for contraception in a rural setting in Pakistan.

**Methods:**

In-depth interviews were conducted with 27 women who were using a LARC method for contraception. Data was managed using NVivo 12 and themes were identified using a content analysis approach to analyze the transcripts.

**Results:**

Four key themes, supported by sub-themes relating to a temporal model, were identified to explain women’s experiences with initiating and continuing use of a LARC. The themes were (i) *Use of trusted networks for information on LARCs*; (ii) *Personal motivation and family support in decision to use LARC; (iii) Choice of LARC methods and access to providers; and (iv) Social and professional support instrumental in long term use of LARC*. Results highlight the significant role of immediate social network of female family members in supporting the women in initiating LARCs and maintaining the method’s use.

**Conclusion:**

This study contributes to an in depth understanding of the decision-making process of women who adopted LARC and maintained its use. Women who proceeded to use an LARC and who persisted with its use despite the experience of side effects and social pressures, were able to do so with support from other female family members and spouse.

## Plain English summary

High proportion of married women do not use an effective contraceptive method and is a public health concern that is associated with unintended pregnancies, unwanted births, and unsafe abortions. This is also associated with increased risks of pregnancy and childbirth-related maternal morbidity. The decision to initiate use and continue the use of contraceptives is influenced by a number of factors. A greater understanding of these factors will inform policymakers, programmers, and other stakeholders to strengthen family planning and other health intervention programs to achieve the SDGs targeted maternal and under-5 child mortality reduction. Thus, the main objective of this study was to understand the social influences in initiating and continuing use of an LARC methods for contraception in a rural setting in Pakistan, using qualitative research. The analysis was done from a total of 27 women, aged 15–49 years, who started the use of long acting reversible contraceptive in the year preceding the study. The findings have an implication for policymakers, programmers, health care providers, and other stakeholders to evaluate and strengthen the provision of different contraceptive methods and improve support provided to women through the family planning programs. The findings of this study strongly recommended the provision of quality counseling on side effects and availability of contraceptive methods, peer support, and support for decision-making regarding long acting reversible contraceptive use.

## Introduction

While annual global population growth rates have declined since the 1960s, population sizes in the least developed countries of Asia and in sub-Saharan Africa have been growing fast and are predicted to grow at substantial levels in the coming decades [1]. Alongside, the prevalence of contraceptive use has increased worldwide [2], however, millions of women in low and middle income countries (LMIC) remain vulnerable to short spaced, unintended pregnancies due to limited access to suitable contraceptives. Recent UN and WHO estimates suggest that more than 220 million women of childbearing age in LMIC are not using any method of contraception [2–4].

The long acting reversible contraceptives (LARC) including implants and intrauterine device (IUD) are highly effective contraceptives. The LARCs are suitable for women of all ages; in comparison, there is significant contraceptive failure with other contraceptive methods (including pill, patch, or vaginal ring), particularly among younger aged women [5]. Moreover, LARCs convey many other advantages for clients in terms of convenience, satisfaction, ease of continuation, likelihood of avoiding unintended/unwanted pregnancy, and non-contraceptive benefits [6–8]. However, LARC use in South Asia accounts for only 2% of the total modern contraceptive methods mix [4, 9]. Barriers for uptake of LARC include issues of access, affordability and insufficient promotion and misconceptions about their effects [10, 11].

Despite evidence from DHS surveys that 98% of the adult Pakistani population have an awareness of at least one modern contraceptive method [12], only 25% of married couples in Pakistan used a modern method of contraception. LARC usage has increased only modestly over the past decade in Pakistan, from 2.1 to 3% [12–14]. Such low uptake of LARCs appears somewhat puzzling in the context of high awareness of modern methods and LARC methods being made available through public sector facilities at subsidized costs [15, 16]. This level of use is lower than in other areas of South East Asia, particularly neighboring Iran, where LARC methods are used by 8% of contraceptive users [17].

Past research exploring Pakistani women’s perceptions about LARCs, especially about IUCDs, showed similar barriers in other countries, including fear of side effects, husband disapproval and religious opposition [18–24]. Women with a higher educational level, employment status and in a more favorable economic position were significantly more likely than other women to use LARCs [25]. Availability and affordability of LARC methods are documented to be barriers to the use of LARCs, particularly among postpartum women [26–28]. There is a need for better understanding the issues affecting LARC uptake, to increase use and create demand for all FP methods using mass media, community health workers and subsidized services.

Previous research on contraceptive use among Pakistani women has focused on assessing reasons for non-use of modern contraceptives, documenting knowledge gaps and access barriers; but it has not investigated in depth, social networks associated with the uptake, and motivations to use, LARCs [23, 29–32]. This study aims to fill this gap and to develop an in-depth understanding of social influences in initiating and continuing use of an LARC methods, including married women’s motivations, information pathways accessed, decision making process, for contraception in rural Pakistan.

## Methods

### Study setting

This study derives from a larger research project that sought to study the FP knowledge and practices among married men and women living in rural Islamabad, Pakistan, conducted in six rural communities. Driven by the study objective, the study population was identified from those rural areas where contraceptive products and services were provided through both public and private sector providers [12]. In the public sector, the Department of Health (DoH) provides services through a network of Reproductive Health Services (RHS) clinics attached to hospitals and the Population Welfare Department (PWD) provide services through Family Welfare Centers (FWCs) in rural areas and Mobile Service Units (MSUs) for hard-to-reach areas. A female community health worker (Lady Health Workers—LHWs), based in each village, have records of each household where they counsel women, provide advice on the benefits of family planning and also provide women a selected range of contraceptives. DoH and PWD clinics provide LARCs at a subsidized cost, such that IUCDs cost Rs. 200 (US$ 20 cents), while implants are provided at no cost.

In the six study areas identified for this research, five Basic Health Units (BHU), one Rural Health Center (RHC), and three Family Welfare Centers (FWC) were operational and providing family planning products (condoms, pills, hormonal injections and IUCD) and services through a medical doctor and licensed midwives (Table 1).

**Table 1:** Population and family planning services in study area

Study area Islamabad capital territory	Population size	LHWs	BHU/RHC	PWD facility
Bhimbar Tarar	6815	10	1 BHU	1 FWC
Bukar	4468	09	1 BHU	1 FWC
Chirrah	14,536	26	1 BHU	–
Tumair	12,173	12	1 BHU	–
Jagiot	7936	22	1 BHU	1 FWC
Tarlai	39,649	33	1 RHC	–

*LHW* Lady Health Worker, *BHU* Basic Health Unit, *RHC* Rural Health Center; *PWD* Population Welfare Department

### Study design

Data examined in this paper includes in-depth interviews with women identified during a broader study of FP knowledge and practices, with 800 married men and women, living in rural communities of the study area [33]. As the objective of this study was to understand factors affecting use of LARC among married men and women in rural communities of Islamabad, a sufficient sample size for undertaking logistic regression modelling was recruited.

### Sampling and recruitment

The respondents for the qualitative study were identified from among the 400 women who participated in a reproductive health and family planning survey conducted in six rural areas of Islamabad [33]. For the survey, married women who were less than 35 years of age and whose youngest child was ≤ 5 years of age were identified by local LHWs, from their household records. Among those women, 47 married women were identified as being a current or past user of LARC and invited for participation in this study. However, after subsequent screening to exclude those who had used a LARC over the past twelve months back and those who had discontinued, 27 respondents fulfilled all criteria. These women were interviewed in their own homes, in the local language (Punjabi Potohari) by the first author (MS) and a research associate (RA); both women and trained in qualitative research methods.

Using a semi-structured, in depth interview guide respondent’s knowledge of contraceptive and FP methods, sources of information, current and past use of contraceptives, reason for using selected method, and experience with the acquired method and its continuing use or non-use, role of social networks and decision-making process on use/non-use of LARC was collected. Although data saturation was achieved after interviews with 12 women, it was decided to interview all women identified to develop insights on differing perspectives. Interviews, lasting 30 to 60 min, were recorded with respondents’ permission, transcribed into English by the RA, verified and finalized by main author (MS), detailing context, where necessary, using notes taken during interviews and field visits. Daily field notes and a reflexive journal were also maintained by the interviewing researcher to record social and interactive nuances observed, as well as to contextualize and corroborate information gathered from interviews.

### Data analysis

Interviews were conducted in the local language and audio-recorded with respondents’ permission. The data were transcribed directly to English by a person fluent in both English and Potohari. MS checked the transcripts against the recordings to ensure accuracy and appended the field notes taken during the interactions. Transcripts were discussed with participants for checking reporting accuracy. The final transcripts were deidentified prior to data analysis using qualitative analysis software program, NVivo 12. The data was analysed using content analysis approach, keeping in mind the context of the study [34]. Research team members undertook an iterative, inductive process of data analysis by independently reading a few of the transcripts to identify meaning units, emerging patterns, and develop a coding structure. The developed codes were then discussed among the research team members to compare and resolve discrepancies. Three transcripts were also shared with two other qualitative researchers for independent coding and verification; differences in interpretation were addressed through discussion and incorporated in the coding structure. The final coding structure, from codes to categories and themes was then used to code all the transcripts. The themes connecting the codes within each category were then identified and are reported descriptively in this manuscript. The Consolidated Criteria for Reporting Qualitative Studies (COREQ) 32-item checklist [35] was followed to ensure reporting consistency.

### Trustworthiness of qualitative data

Methodological rigor for this research was achieved through first author’s prolonged engagement with the respondents, respondent validation, maintaining a chronological audit trail, team debriefings and triangulation for data analysis, with the co-authors (SH and RJ) [36, 37]. Some of the data were also collected, transcribed and coded by the Research Assistant (RA), to ensure that the first author’s (MS) professional orientation as medical doctor and public health researcher did not impact on data collection approach and analysis process. Probes were used to explore the responses and provide context for the information shared. Throughout the process of data collection and analysis, ongoing critical reflection, peer discussions, as well as respondent validation ensured interpretative accuracy of the results. Contextual details of the study site were captured through field notes, pictures of some areas, and a reflexive journal, all of which were used to provide a holistic description of the study area setting and participants. Neutrality of findings was maintained through debriefings within in the team, combining perspectives of the entire research team.

### Ethical approval

Ethical approval for the study was obtained from Human Research Ethics Committee (HREC) of University of New South Wales and National Bioethics Board (NBB), Pakistan. Informed consents were solicited, and participants were assured of confidentiality of information shared; initials have been used throughout to protect participants’ identities.

## Results

### Participant characteristics

The age of the 27 participants ranged from 20 to 33 years and had 2 to 4 children each. 55% had attended primary school only and none were formally employed, and all were housewives. Most of the women interviewed had used other types of modern contraceptive methods prior to using an LARC, with 63% using condoms. Currently 23 (85%) were using IUCD (both copper and hormonal devices) as LARC.

### Qualitative study findings

Women’s experiences with initiating and continuing use of an LARC are explained under four themes, (i) *Use of trusted networks for information on LARCs*; (ii) *Personal motivation and family support in decision to use LARC; *(iii)* Choice of LARC methods and access to providers; and *(iv)* Social and professional support instrumental in long term use of LARC,* supported by sub-themes.

**Fig. 1:**
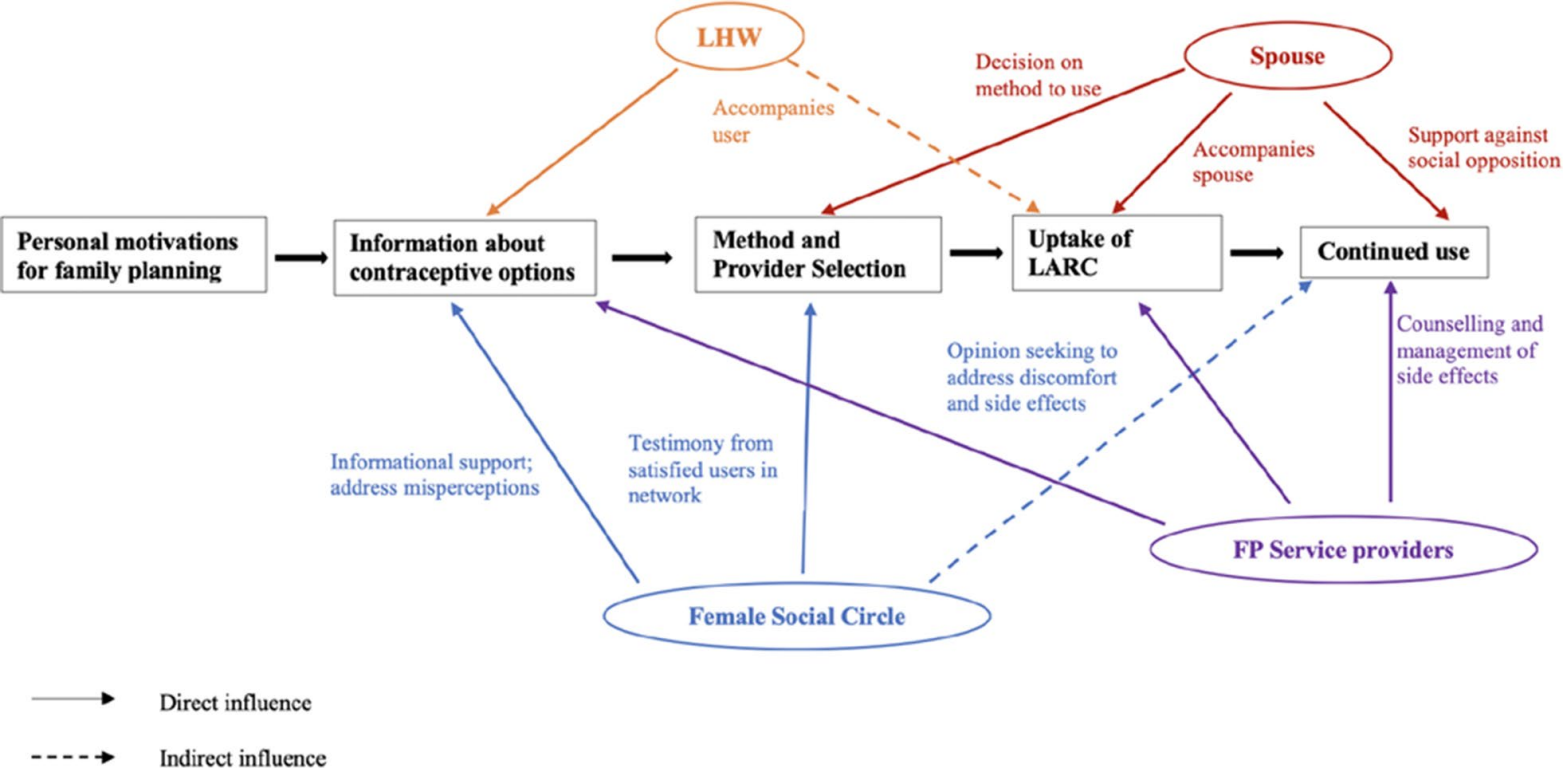
Conceptual model of women’s decision making about LARC use in Pakistan; (social groups identified in different colors. Legend: *LHW* Lady Health Worker; *FP* Family Planning)

The figure below (Fig. 1) shows a conceptual model of LARC use decision making, which emerged inductively from the data. It illustrates women’s staged decision-making process about LARC uptake and use, providing a framework for understanding important influences at each stage. The model identifies the social influences operating at each stage, provided social relationships and personal interactions. As shown in the model, women are interested in initiating use of a contraceptive due to personal motives. They then proceed to gathering information about methods and providers from trusted sources, including female relatives and local LHWs. Experiences of satisfied users lessened women’s reservations about LARC methods; any lingering doubts were also addressed by skilled providers, helping them overcome this barrier to adopting use of a LARC. Women then proceeded to deciding the type of method to use, with support from their spouse, who also accompanied them to the provider. After initiating use of a LARC method, ongoing support of spouse, and skilled providers gave women the confidence to continue use of this contraceptive method.

#### Use of trusted networks for information on LARCs

Women sought to obtain information about different types of contraceptives, particularly about LARCs from different sources when they were contemplating using an LARC.

### Communication networks

Most stated that their source of information about contraceptives in general and LARCs, were predominantly from female relatives (mothers, sisters, aunts, cousins). In all cases, these female relatives had also used contraceptive methods, including LARCs, mostly IUCDs. It seems a common practice, even normative, for married women to share their experiences of contraceptives with other women in their relatives network.

My older sisters are married, and they would talk about family planning methods; so, I knew from them that there were things you can use for having [a] gap in children. One of my sisters has been using the ring (IUCD), for 5, 6 years now. When we have a health problem, we first ask our mother or sister, sometimes mother-in-law also … [Respondent 3, 30-year-old]

In cases where, had medical interventions (e.g. cesarean section) they received information about LARC methods by their obstetricians and nurses. However, they consulted with family members about such advice:

Doctor said that I should have a gap of at least 3 years between children, because of operation (C-Section). She told me that the hospital has Family Planning clinic and I can get the challah (IUCD) and capsule (implant) from there, after my post-partum period (chillah) ends. I asked my mother about this; she and my sister had also used challah (IUCD) and they also told me to start using something … [Respondent 6, 26-year-old]

### Verifying information from experienced users and professionals

Various misconceptions about both LARC methods were common. Many mentioned knowing someone who had experienced adverse side effects of IUCD (such as perforation through uterine wall and migrated to the abdomen or kidney) or they knew women who had become pregnant while using these contraceptive methods:

… My younger sister also had it, but she conceived with the tube (IUCD) inside and had her daughter same time as my son [Respondent 12, 24-year-old]

Information fueling these misconceptions was reportedly shared through social networks, where sometimes the use of a particular LARC was discouraged, even by the LHW:

…. I have heard that the rods (implant) can stop menstrual cycles and cause infertility, if used for a long time; so, I decided to use the ring (IUCD) [Respondent 18, 33-year-old]

It was also found that in addition to seeking information from female relatives, women sought advice of local LHWs, which was sometimes biased.

… My sister-in-law was using the capsules (Implant) so I asked her about her experience with using it; she said it was easy to use and she had no problems. Then I asked Baji (LHW) about it but she said the capsules (implant) can cause problem in the woman’s system, like I can stop having my periods or I can have too much bleeding or spotting. She also told me that some women had to take treatment before they could become pregnant again. She said better to get the tube (IUCD), so I decided to get that [Respondent 16, 27-year-old]

Some of the women who had experienced regular interactions with a doctor or a nurse at ante natal clinics and hospital following cesarean section were more likely to take advice from their doctor or a nurse about starting the use of an LARC.

… both my children were born by operation (C-Section); after my second operation, doctor told me to use a family planning method. She said they have capsules, which they put in the arm … [Respondent 14, 27-year-old]

### Personal motivation and family support in decision to use LARC

#### Personal circumstance motivates decision to use LARC

Women’s concerns for their own or their child(ren)’s health was found to be a primary determinant in their decision to start using an LARC method, especially when traditional methods failed.

… the LHW told me that I will not get pregnant if I breastfeed the baby, but I became pregnant again when my daughter was 7 months old. Both my children were born by c-section operation. It’s very difficult to care for two young children, especially after operation so I thought I will have a gap of four, five years before having another baby [Respondent 11, 25-year-old]

A common factor among all the women who opted for LARC was the prior use of other modern contraceptive methods; condoms had been used by all, while some women had also used the contraceptive pill and injections. Women reported the desire to start using a long-term method, citing their husband’s reluctance to use condoms as well as their own difficulties with adherence to the regular taking of pills and receiving injections:

… we used Saathi (condoms) a few times; but my husband did not want to use the condoms and we did not want another child for some time, so I decided to get the tube (IUCD) [Respondent 24, 26-year-old]

Capsules (Implant) are good; better than injection or the tube (IUCD). With the tablets, you have to remember to take it every day and sometimes you can forget to take it on time. Injection also same problem [Respondent 7, 30-year-old]

#### Support of spouse in decision making

All the women shared that they had discussed child spacing with their husbands and had together considered the long-term contraceptive methods available, the duration of each, and the number of children they wanted. Since there is a cultural norm in Pakistan of seeking the approval of one’s husband in such matters of having an LARC.

My husband is also in favor of having gap in children. I told him about the tube (IUCD) and I can have it for five years or ten years; he said to get the 5 year one [Respondent 6, 26-years-old]

I talked to my husband about it (IUCD); he told me to get the IUCD when we had our first daughter (after two sons), but I wanted to have another girl before getting this (IUCD) [Respondent 30, 28-years-old]

#### Importance of support from female family members in decision making process

The supportive role of an older female relative, most typically mother, mother-in-law or elder sister was significant for respondents in choosing an LARC method and even influencing the spouse.

Both my children were born with gap of just six month and I wanted to have some gap before another child. So, I talked to my husband about this challah (IUCD) method and told him that there is option for 5 years and 10 years; but he did not allow me. Then I asked my mother-in-law and she told him to give permission to get the challah (IUCD) [Respondent 34, 24-years-old]

This supportive role of older female relatives was also observed in situations where a female relative (mostly a mother or sister-in-law) of the respondent expressed support for the decision of the respondent, citing reasons of low income, and high costs of living:

…. I got the IUCD after I had two children … (Respondent 9, 26-years-old); (MiL joined in discussion) …. after her second daughter, I told her to get the tube (IUCD); I also told my daughter the same thing; for me, both (daughter and daughter-in-law) are equal and having a boy or girl is Allah’s decision. Nowadays, everything is so expensive and raising children properly in limited income is difficult

### Choice of LARC method and access to providers

After having decided on starting use of an LARC, women selected the appropriate LARC method and provider. Both these decisions were driven by the experiences of trusted members in their social networks.

#### Testimonies of LARC users

Respondents sought trusted female family members to enquire about appropriate LARC methods and providers. Their experience with either of the two LARC methods influenced respondents in deciding the choice of method and provider.

I got the capsules (implant) because two, three of my relative got the challah (IUCD) and they suffered from a lot of health problems. They said not to get it (IUCD) [Respondent 27, 29-year-old]

Women also preferred getting advice about LARC methods from providers recommended by a trusted relative:

… I asked my cousin also about the tube (IUCD), I knew she had it; she said she got it from NIH. So, I went there after the post-partum period ended [Respondent 17, 27-year-old]

#### Endorsement by professionals

LHWs are generally trusted for advice about women’s health, especially reproductive health, as well as their knowledge of contraceptive methods and their recommendations about suitable LARC providers. Some women also relied on their local LHW to accompany them to the selected LARC provider. Respondents invariably approached the LARC provider who was recommended by either a relative or the local LHW or their obstetrician:

I asked Baji (LHW) about the capsules, she also said it is good and very easy to use. She went with me also, to Poly Clinic to get the capsules …. [Respondent 18, 28-year-old]

#### Ease of access to providers

None of the respondents indicated access issues about LARC use, including distance to provider or affordability. If the chosen provider was at some distance from their residence, respondents generally travelled via public transport, often accompanied by a female relative, local LHW or spouse/husband:

I went to Poly Clinic on the local transport from here, with my sister-in-law (bhabi). She had also got hers from there, so I asked her to go with me (user) [Respondent 5, 29-years-old]

…. she (mother-in-law) took me to N Baji’s (LHW) house and asked her to go with us to the doctor in Falahi Markaz. My mother-in-law said to get the tube (IUCD) which is for 10 years and I had that placed from there. [Respondent 13, 20-years-old]

The respondents mentioned the availability of LARC methods at the local public sector health facilities, including local primary health care and Population Welfare Department clinics. The respondents had easy access to these facilities.

…. I had the tube (IUCD) placed from Falahi Markaz here in our village; other women in the village also got it from there. It’s also near my house and was easy for me to go to … [Respondent 7, 28-year-old]

### Social and professional support is instrumental in continuing use of LARC

Women who were using an LARC were generally satisfied with the method and planned on continuing use for spacing or having reached their desired number of children, intended to continue it.

#### Spousal and family support for continued use of LARC

In continuing to use an LARC, the level of support from one’s spouse and social networks was found to be important. Women countered any negative social criticism when they had the support of their husband. They considered women who did not have support of their husband to be helpless and vulnerable to multiple pregnancies interspaced with very short time intervals:

I told my neighbor about this method as well, but she said her husband did not agree and now the poor thing (bechari) is pregnant again, with her third child. It’s all about the husband’s support in using the contraceptive methods! People say all kinds of things to me, but I say if my husband is with me on this, then I don’t care what you say. [Respondent 2, 24-years-old]

Women reported seeking advice from female family members about side effects and any discomfort experienced. They were generally advised by female family members not to worry about side effects as they were told these effects would settle over time. Women were reportedly reassured by such supportive advice, especially those who were using an LARC for the first time:

Few days after I had my ring (IUCD) placed, I felt some irritation and burning. I told my sister about it and she said it happens for some time, but it will go away in some days, so don’t worry about it. She said she also had it after she got hers (IUCD) but then it was fine. (I: When did she get her IUCD?) She has had it for four, five years now; actually, she had told me to get it after my daughter was born [Respondent 19, 27-years-old]

#### Seeking counselling and timely treatment from health care providers

Normality in monthly cycles was considered important by all participants; a deviation from routine was worrisome, leading to fears of this being an imminent sign of developing side effects. Respondents shared that their initial experience of some side effects like pain or some spotting or of heavier menstrual bleeding than normal, was alarming for them, and they were anxious about developing more serious health problems. Women reported dreading the thought of having the LARC removed, as they did not want another child at that time.

Initially I did not have any problem; but after about three months, I had some spotting two or three times. That really scared me, because I had heard of other women who had similar problems. But then it settled with the medicine doctor had given me; and now I’m afraid to share with anyone that all is ok for fear that I might develop some side effect again! [Respondent 35, 32-year-old]

Both current and past LARC users appeared to have been counselled well by their provider about the method and any expected side effects. As a result, when women initially noticed side effects, they reported not seeking immediate care in anticipation of the side effects resolving or subsiding with time. LARC providers were generally approached when side effects continued or increased in intensity:

After the IUCD, I had prolonged monthly bleeding, like it was 8 or 9 days and I was still having bleeding. So, I went back to the Falahi Markaz and doctor there gave me a medicine to take for one week, told me not to worry about this …. I took the medicine and now it’s been almost a year and thank God I have not had any problem again! [Respondent 16, 28-year-old]

## Discussion

This study set out to investigate social influences on women’s decision making about LARC use and provides an insight on women’s motivations, information pathways accessed, and decision-making process for LARC uptake in rural Pakistan. Where previous studies examined barriers to and discontinuance of LARC [18, 23, 30, 38], this is the first study offering insight on key people and their various roles in influencing and supporting women in their decision to use and continue to use LARCs. The study identifies four themes in the pathways of married Pakistani women choice to take up an LARC and continue its use: (i) *Use of trusted networks for information on LARCs*; (ii) *Personal motivation and family support in decision to use LARC; (iii) Choice of LARC methods and access to providers; and (iv) Social and professional support instrumental in long term use of LARC.* Each of these themes fit a temporal sequence in adoption of LARC but cannot be taken as stages as often there is overlap between each of them and are discussed together. Policy implications of the findings are also discussed in the context of current policy in Pakistan.

The effects of social networks and inter-personal communication on shaping women’s attitudes about family planning and contraceptives is well established in social networks research, which has shown to have had effect on contraceptive use behavior [39–42]. Research evidence from regional countries, similar in context to Pakistan, have also reported that women’s communications with female family and social network members influenced their knowledge and attitudes towards contraceptive use in family planning [43–45]. However, in the current study, the “trusted” social network was comprised mainly of female relatives rather than friends or “experts” external to the family. This difference may be attributable to the culture of consanguineous marriages, norms of socio-cultural interactions limited to extended family, and restrictions on women’s mobility without a male or an older female escort in Pakistan [32, 46]. Conversations about contraceptive use are rare and unconventional with non-family members of one’s social network and may contravene established social and cultural norms in Pakistan. As such, it remains to be seen whether the diffusion of LARCs could be enhanced through extended female social networks that have used LARCs or whether such conversations will generally be confined to female family members and FP providers.

Prior qualitative studies have shown that Pakistani women have had some reluctance to use contraceptives, especially LARCs, because of myths and misconceptions about long term side-effects, which have largely been driven by rumors and shared through social communications [23, 29]. The current study also found such occurrences, some which were spread by providers, specifically the LHW. However, what this study found was that women who adopted LARCs, were able to discuss this with trusted female family member. The shortcomings of the LHWs may be because of gaps in their knowledge and deficits in counselling skills, arising from limited training as community health workers, as well as a lack of formal training in health care, which were factors identified in an external evaluation of the LHW program [47, 48]. The National Population Policy [49] identifies LHWs as key personnel to provide counselling to reduce the unmet need for contraception on Pakistan. It would be fair to surmise that uptake will increase when a sufficient cohort of older female family members with experience of an LARC are available to counter these myths and misconceptions.

The study findings suggest that increasing Pakistani women’s access to satisfied LARC users, within peer networks have the potential to support women in their decision making for uptake and use of LARCs. Experience of neighboring countries with interventions for increasing use of LARCs may also offer ideas for implementation in Pakistan. For example, in countries such as India, Nepal and Bangladesh, where women are influenced in their decisions about maternity care and contraceptive use by other women in their social networks [50–53], those countries have introduced behavior change communication programs. Such programs are aimed at improving uptake of contraceptive methods through joint discussions with women’s groups comprising younger married women alongside other experienced women including mothers and mothers-in-law [39, 54]. However, it remains unclear whether such strategies would suit the cultural conditions in Pakistan.

Husband endorsement and their shared role in family planning decision-making was acknowledged by the women as necessary for their LARC uptake as well as for their continued use of an LARC. Participating women told us how they tried to influence their spouses to arrive at a joint decision and when such a joint decision was not forthcoming how they relied on more senior women in their husband’s family to influence their husband. The pivotal role of spousal support in contraceptive choice is highlighted in research from Pakistan which has explored men’s roles in family planning and have promoted couple counselling based on that premise [40, 41, 55]. Previous qualitative research which explored men’s family planning attitudes and information needs, has also highlighted the importance of involving husbands in family planning discussions and the need to establish forums for men as a way of raising awareness, addressing myths and concerns, and advocating for supporting women’s use of LARC [40, 42]. While husband endorsement to use a modern contraceptive was vitally important for the women in the current study, the greatest influence on their decision to use an LARC appeared to be older female family members.

### Limitations and strengths

As the study concentrated only on current users of LARC, the views of non-users and those who discontinued was not available for a nuanced comparison. However, given issues of non-use and discontinuation have been looked at in other studies, this study sought a unique contribution. Another limitation is the relatively small number of hormonal Implant users, as compared to the IUCD users. However, as per the PDHS 2017–2018, in the overall contraceptives mix use in Pakistan, Implants are used by only 0.4% women and this disproportion is also reflected by our study sample. As with any study based on convenience samples, caution is needed before generalizing the findings beyond the sample group.

A key strength of the study was identification of respondents for the qualitative research, subsequent to a community survey; this allowed triangulating the qualitative findings such as contraceptive knowledge and practice gaps, and the role and influence of peers in promoting the use of contraceptives. In addition, these study findings have importance beyond the sample studied as they contribute valuable insight for policy makers and program managers for family planning services development.

## Conclusion

Much of the existing body of research on women’s use of LARCs in Pakistan is based on surveys, measuring women’s knowledge and use of all types of contraceptives, including LARCs. This study was unique in exploring in depth the decision-making process of women who adopted LARC and maintained use. Results have highlighted the significant role of their immediate social network of female family members as the key influencers of decisions at all points of the process. Women who proceeded to use an LARC and who persisted with its use despite the experience of side effects and social pressures, were able to do so with support from their spouse and other female family members. Study findings have to be considered in light of national policy of using the LHW to motivate use of modern contraceptives. Creating informed demand for LARC in Pakistan requires more intensive community-level efforts. It is proposed that as more women in an area adopt LARCs, access to satisfied users within social networks should be facilitated, which can increase and create a more favorable environment for uptake of LARCs, expanding women’s choice.

## Data Availability

Data used in this study can be made available, in an anonymized format.

## Abbreviations

ANC: Antenatal care
BHU: Basic Health Unit
CPR: Contraceptive Prevalence Rate
DHQ: District Headquarters Hospital
DHS: Demographic and Health Survey
FGD: Focus Group Discussion
FP: Family planning
FPAP: Family Planning Association of Pakistan
FWW: Female Welfare Worker
ICT: Islamabad Capital Territory
IDI: In-depth interview
IMR: Infant Mortality Rate
IUD: Intra Uterine Device
KPK: Khyber Pakhtunkhwa (Province)
LHS: Lady Health Supervisor
LHW: Lady Health Workers
LMIC: Low- and middle-income country
mCPR: Modern Contraceptive Prevalence Rate
MNCH: Maternal and Child Health
MMR: Maternal Mortality Ratio
PDHS: Pakistan Demographic and Health Survey
PHC: Primary Health Care
PWD: Population Welfare Department
RH: Reproductive Health
RHC: Rural Health Unit
SDGs: Sustainable Development Goals
STI: Sexually transmitted infection
TBA: Traditional Birth Attendants
TFR: Total Fertility Rate
